# Between war and pestilence: the impact of armed conflicts on vaccination efforts: a review of literature

**DOI:** 10.3389/fpubh.2025.1604288

**Published:** 2025-07-01

**Authors:** Fausto Ciccacci, Emanuela Ruggieri, Paola Scarcella, Stefania Moramarco, Mariachiara Carestia, Daniele Di Giovanni, Loredana Andreea Silaghi, Anna Maria Doro Altan, Stefano Orlando

**Affiliations:** ^1^Department of Biomedicine and Prevention, Tor Vergata University of Rome, Rome, Italy; ^2^LUMSA University, Rome, Italy; ^3^Link Campus University, Rome, Italy

**Keywords:** vaccination, conflict, war, immunization (vaccination), prevention, refugee health, humanitarian health

## Abstract

Armed conflicts profoundly undermine vaccination efforts, disrupting healthcare systems, displacing populations, and enabling the resurgence of vaccine-preventable diseases (VPDs). This narrative review explores the relationship between conflict and immunization coverage through an analysis of 18 studies across diverse regions, including Syria, Nigeria, Afghanistan, Iraq, and Ukraine. Evidence reveals that countries affected by war account for a disproportionate share of global polio and measles cases, often due to damaged infrastructure, interrupted cold chains, and vaccine hesitancy exacerbated by political instability and misinformation. Refugee populations, particularly children, face additional barriers such as poor access, low vaccine literacy, and economic hardship. Despite these challenges, innovative responses have emerged: mobile vaccination teams, negotiated access with armed groups, integration with other humanitarian services, and the use of digital tracking technologies have helped mitigate immunization gaps. However, these are often temporary solutions. Sustainable vaccination coverage requires not only emergency interventions but also long-term conflict resolution. Ceasefires and humanitarian pauses have allowed short-term immunization campaigns, yet their effectiveness is limited without durable peace and systemic rebuilding. The findings highlight the need for coordinated global efforts to protect immunization programs in conflict zones and to uphold vaccination as both a public health priority and a human right.

## Introduction

The intersection of vaccination and armed conflict has deep historical roots. Some historians suggest that Napoleon gained a strategic military advantage in his European campaigns due to variolation (1). While the accuracy of this claim is still debated, it highlights how the connection between war and immunization is not new. In modern conflicts this relationship has changed, as wars disrupt healthcare infrastructure, displace populations, and hinder vaccine distribution, leading to the resurgence of preventable diseases (2, 3). Misinformation and political instability fuel vaccine hesitancy, while armed groups sometimes obstruct immunization efforts for strategic purposes (4). These effects extend beyond war zones, as displacement and inadequate healthcare access increase the risk of cross-border outbreaks (5).

Ensuring vaccine access in conflict-affected regions is both a public health necessity and a human right (6). Some countries have sustained immunization programs even during conflicts and political instability through innovative delivery strategies, targeted investments, and international collaborations (7). Strengthening these efforts is crucial for long-term global health security.

Despite growing evidence from individual conflict zones and regional analyses, there is no comprehensive synthesis of these impacts across countries and age—to date, the literature lacks a global narrative or systematic review integrating cross-country data on conflict-related immunization disruptions (8). Furthermore, immunization is often treated as a secondary concern during wars and humanitarian emergencies (9). Major evidence gaps remain unaddressed, including missing immunization data from many conflict-affected regions (due to insecurity and surveillance breakdowns), the underrepresentation of certain vaccine programs (such as adolescent HPV or adult booster vaccinations) in research and policy discussions on conflicts (10), and minimal attention to long-term effects—for instance, how cohorts of under-vaccinated individuals emerging from protracted conflicts may sustain outbreaks and impede disease control even after hostilities cease (11).

The aim of this narrative review is to synthesize the global evidence on the impact of armed conflict on vaccination coverage across all age groups and routine immunization programs, highlighting neglected issues and informing future policy and research priorities.

### Review approach

To explore the impact of armed conflicts on vaccination coverage, we conducted a narrative review of the literature. A search was performed on PubMed using the following query: (“Vaccination”[Mesh] AND “Armed Conflict”[Mesh]).

**Figure 1:**
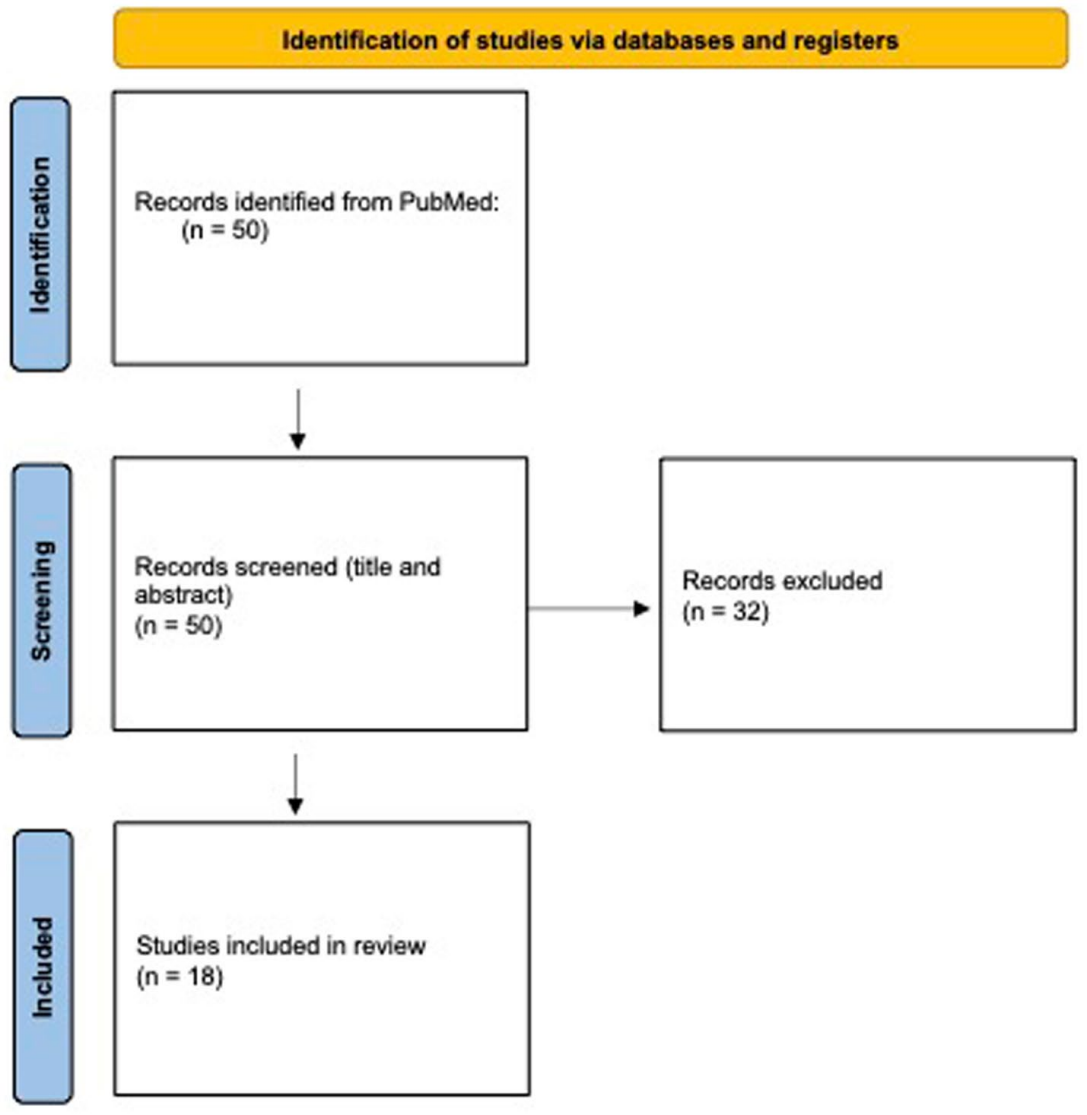
PRISMA 2020 flow diagram summarizing the study selection process. Source: Page et al. (33). This work is licensed under CC BY 4.0. To view a copy of this license, visit https://creativecommons.org/licenses/by/4.0/.

The search, conducted in December 2024, yielded 50 results. Titles and abstracts were screened by two independent reviewers, who assessed the relevance of each study to the research question. Studies were included if they examined disruptions in immunization campaigns, reductions in vaccine coverage, or the re-emergence of vaccine-preventable diseases in conflict settings. Studies that did not specifically address these aspects or lacked empirical data were excluded. After title and abstract screening, 32 articles were excluded due to irrelevance to the review topic, and 18 articles were included in the narrative review (Figure 1).

The selected studies represent a diverse set of conflict-affected regions, including Nigeria (2 studies), Afghanistan (2 studies), Iraq (2 studies), Ukraine (2 studies), the Democratic Republic of the Congo (2 studies), Pakistan (1 study), Syria (1 study), Poland (migrants from Ukraine, 1 study), Germany (refugees from Syria and Afghanistan, 1 study), Gaza (1 study), Lebanon (1 study), Sudan (1 study), one paper with global perspective, and a broader review covering 16 countries (1 study). Table 1 summarizes the key characteristics and findings of these studies.

**Table 1:** Studies included in the analysis.

Study	Country	Disease(s) studied	Main impact of the conflict
Baatz et al. (21)	Syria	Measles, polio	Importance of international support to local organizations for vaccination governance, reductions in vaccination coverage, increase in epidemics
Nnadi et al. (12)	Nigeria	Polio	Reactivation of the virus in areas inaccessible due to insecurity
Cousin (11)	Afghanistan	Polio	Logistical barriers for vaccinations, increase in cases
Lafta and Hussain (13)	Iraq	Measles, Hepatitis B, TB	Measles outbreaks recurring at 4/5 year cycles, increase in Hepatitis B and TB
Debate (14)	Ukraine	Measles, polio	Increase in measles cases and reduction in vaccination coverage due to logistical difficulties, conflicts and false information
Nakkazi (23)	Democratic Republic of Congo	Ebola	Slowed recognition of epidemic due to conflicts
Lewtak et al. (16)	Poland	Diseases preventable by vaccination	Difficulty in accessing prevention and vaccination services due to language barriers and misinformation
Grundy and Biggs (8)	16 countries	Measles and Polio	Conflict undermines vaccination through destruction of infrastructure, insecurity, staff shortages, and access barriers, leading to VPD outbreaks and health inequities
Checchi (12)	Nigeria		Crude mortality rates in conflict areas are about double national rates
Edmond (19)	Afghanistan	Essential care and vaccinations	Greater effectiveness of on-site vaccinations and essential care delivered by mobile teams compared to care and vaccinations in central facilities
Ngo et al. (9)	Afghanistan, Pakistan, Nigeria, Somalia…	Vaccine-preventable diseases	Negative effect of wars: polio re-emerges in areas where it was eliminated and vaccination coverage collapses
Wells et al. (24)	Republic of Congo	Ebola	Reduction of vaccinations and study of a model to evaluate the speed of response to the development of an epidemic
Fozouni et al. (15)	Germany	Vaccine-preventable diseases	Greater coverage with vaccinations carried out directly in the refugee camp
Mansour et al. (17)	Lebanon	Polio, dtp	Discrepancy between coverage of Lebanese children versus Syrian refugees
Lam et al. (18)	Iraq	Cholera	The use of vaccination must complement core public health interventions as a comprehensive response to epidemics
Mahase (20)	Palestine	Polio	Polio cases and vaccination during a truce
Javed et al. (22)	Pakistan	Whooping cough and parapertussis	Whooping cough outbreak due to non-adherence to vaccination campaign and difficulty in reaching war-affected areas
Pham (20)	Sudan	Measles, tetanus	Evaluation of the quality of health interventions, reduction of measles coverage and increase of tetanus coverage

### Insights from the literature review

The analysis of the papers has shown that the impact of armed conflicts on healthcare systems, particularly on vaccination coverage, represents a critical challenge for public health. The fragility of healthcare infrastructure, disruptions in immunization programs, and logistical difficulties in reaching affected populations contribute to declining vaccination rates, exposing communities to the risk of outbreaks of preventable diseases.

### Impact of armed conflict on vaccination coverage: a global analysis

Armed conflicts pose a severe threat to immunization programs, leading to substantial declines in vaccine coverage and increased susceptibility to vaccine-preventable disease (VPD) outbreaks. A systematic analysis conducted across 16 conflict-affected countries found that these nations, despite comprising only 12% of the global population, accounted for 67% of global polio cases and 39% of global measles cases between 2010 and 2015. Furthermore, 14 of these 16 countries recorded diphtheria tetanus toxoid and pertussis (DTP3) coverage rates below the global average of 85%, with some experiencing sharp declines following the onset of armed conflict (8). These findings align with global estimates indicating that over two-thirds of unvaccinated children live in conflict-affected settings, demonstrating the direct correlation between war-related disruptions and immunization failures (9).

### Country-specific case studies: Nigeria, Iraq, and Ukraine

The case of Nigeria highlights the intersection of conflict, forced displacement, and polio resurgence. In Borno state, Nigeria, an area affected by instability since 2013, polio vaccination campaigns were obstructed due to inaccessibility of large portions of the population. As a result, four new wild poliovirus (WPV1) cases were detected in 2016, 2 years after Nigeria had been declared polio-free. These cases were identified in children from conflict-affected and hard-to-reach areas, with genetic analyses suggesting prolonged undetected transmission due to low immunization rates and gaps in surveillance (12). Similarly, in Iraq, the national measles immunization rate declined to 75%, well below the 95% coverage needed for herd immunity, contributing to outbreaks in 2008/2009 and again in 2014. Polio vaccine coverage was also compromised by frequent lack of energy, which disrupted the cold chain necessary for vaccine storage (13). In Ukraine, the ongoing conflict since 2014 exacerbated an already declining vaccination trend. Measles, mumps, and rubella (MMR) vaccine coverage fell from 95% in 2008 to just 31% in 2016, making Ukraine the epicenter of the largest measles outbreak in Europe. Additional factors such as disinformation campaigns and vaccine hesitancy further contributed to the crisis (14).

### Comparative analysis of refugee populations: Germany, Poland, and Lebanon

The impact of conflict on vaccination is illustrated in studies on refugee populations, where displaced children frequently exhibit lower vaccination rates than host community children. In Germany, a study of Syrian refugees found disparities between children residing in different types of refugee accommodations in Berlin (15). In Tempelhof, where in-camp vaccination services were available, 27.8% of children under 5 years were fully vaccinated, while in Neukölln, where access to vaccines depended on external health services, 93% of children were either partially vaccinated or had not received any vaccines at all. In Poland, despite policy provisions granting free access to routine vaccinations for Ukrainian refugee children, coverage remained suboptimal. A study identified key barriers such as language difficulties, low vaccine literacy, economic instability, and concerns over vaccine safety, contributing to incomplete immunization schedules (16). Notably, only 31% of Ukrainian children in Poland in 2016 had received the second MMR dose, significantly below the required 95% for effective herd immunity. Similarly, in Lebanon, where the influx of Syrian refugees has placed additional strain on healthcare infrastructure, a district-based immunization survey found that vaccination coverage was higher among Lebanese children than Syrian refugees, with disparities particularly pronounced for later vaccine doses (17). For instance, while 90% of Syrian children received the first pentavalent vaccine dose, many failed to complete the full schedule, indicating a high dropout rate.

### Strategies to overcome vaccination barriers in conflict zones

Armed conflicts significantly disrupt routine immunization programs, necessitating innovative strategies to ensure vaccine delivery in war-affected areas. One of the most effective approaches has been the reliance on non-governmental organizations (NGOs) to fill healthcare gaps where governmental systems have collapsed. For example, in Afghanistan, 91% of provinces outsourced healthcare services to NGOs, ensuring vaccine distribution in unstable regions, while in Myanmar, NGOs played a critical role in reaching areas controlled by non-state actors. In contrast, the Democratic Republic of the Congo (DRC) lacked a national strategy to vaccinate children in conflict zones, depending entirely on international organizations to manage immunization efforts (8).

To circumvent security risks, many countries have employed mobile and emergency vaccination campaigns. In Sudan, polio vaccination teams adopted a “hit-and-run” approach, entering insurgency-affected areas to immunize children before retreating to safety. Similarly, in Iraq, a 2015 cholera outbreak prompted an emergency vaccination campaign in internally displaced persons camps, achieving an 87% two-dose coverage rate, despite logistical challenges (18). A unique monitoring technique, Lot Quality Assurance Sampling, was implemented in West Darfur, Sudan, enabling real-time assessment of immunization programs and leading to a significant increase in tetanus toxoid vaccine coverage from 47.2 to 69.7% over 19 months (19). Meanwhile, in Gaza, the World Health Organization emphasized the necessity of a seven-day ceasefire to facilitate the vaccination of 640,000 children at risk following the detection of poliovirus in wastewater (20).

An alternative strategy has been negotiated access with armed groups, allowing immunization teams to operate in conflict zones, as reported by Grundy and Biggs (7). In Sudan, local mediators and UN agencies brokered agreements with non-state actors to permit vaccination teams access to contested areas. Similarly, in the Central African Republic, multinational peacekeeping forces provided security escorts for vaccine distribution, mitigating risks to healthcare workers. In Somalia, “Child Health Days” combined vaccinations with nutrition services and infection treatment, while in South Sudan, UNICEF and the World Food Programme launched a Rapid Response Mechanism, pairing food distribution with immunization efforts to maximize outreach in hard-to-reach areas.

Advancements in digital tracking and logistics have also played a crucial role in overcoming operational challenges. In Northwest Syria, an initiative to digitize vaccination records aimed to improve immunization tracking, although financial constraints limited its full implementation (21). In Iraq and Pakistan, GPS tracking of vaccination teams helped enhance coverage in volatile regions by ensuring systematic outreach and reducing missed communities (18, 22).

Overall, these diverse strategies highlight the adaptability required to maintain immunization programs in war-affected areas. While mobile vaccination teams, negotiated access, integration with other health services, and digital innovations have shown promise, sustained success hinges on international collaboration, flexible funding, and context-specific interventions tailored to the unique challenges of each conflict zone.

### The role of ceasefires and conflict resolution in vaccination efforts

While targeted interventions such as mobile vaccination teams, negotiated access, and integrated immunization campaigns have proven effective in mitigating the impact of conflict on vaccine coverage, the fundamental barrier to sustainable immunization remains the continuation of hostilities. Only the cessation of conflict can restore the stability needed for the long-term rebuilding of healthcare systems and routine immunization services. Without peace, any vaccination effort remains temporary, subject to the volatility of war, and ultimately insufficient to prevent large-scale outbreaks of vaccine-preventable diseases.

Recognizing this, international organizations have increasingly advocated for temporary ceasefires and humanitarian pauses to facilitate emergency immunization efforts. In Gaza, following the detection of poliovirus in wastewater, the WHO and UNICEF called for a seven-day ceasefire to vaccinate 640,000 children under the age of 10. The WHO emphasized that such pauses are essential for allowing families to reach healthcare facilities safely and enabling vaccinators to operate without risk. However, the agency also warned that a permanent ceasefire is the only viable solution to ensure long-term public health security in the region (20).

The use of negotiated truces for immunization has precedent in multiple conflict-affected regions. In Nigeria, Sudan, and the Central African Republic, combatants have temporarily suspended fighting to allow for polio and measles vaccination campaigns, particularly in areas under the control of non-state armed groups (8). Similarly, during the Ebola outbreak in RDC, humanitarian organizations secured agreements for safe access to at-risk populations, enabling limited vaccination efforts despite ongoing violence (23, 24).

While localized ceasefires and humanitarian pauses provide short-term opportunities for vaccination in conflict settings, they do not replace the need for a lasting resolution to hostilities. The WHO has repeatedly stressed that while temporary immunization campaigns can prevent immediate disease outbreaks, only lasting peace can guarantee the conditions necessary for routine immunization programs to function effectively. Until conflicts are resolved, millions of children in war-torn regions will remain at risk, highlighting the urgent need for global diplomatic efforts to address both the health and humanitarian crises perpetuated by armed violence.

## Discussion

This review confirms that armed conflicts severely disrupt immunization systems, causing drops in vaccine coverage and surges in VPDs. Though representing a small portion of the global population, conflict-affected countries bear a large share of polio and measles cases—nearly 70% of global polio cases (2010–2016) occurred in such regions (9), and two-thirds of unimmunized children live in unstable countries (25). The Middle East, particularly, has suffered multi-level healthcare disruptions due to protracted wars, leading to destroyed facilities, displaced staff, and collapsed cold chains (26). This degradation has enabled the return of controlled VPDs: polio reemerged in Syria after 14 years, and measles outbreaks surged in Iraq and Ukraine. These trends echo past events like the Yellow Fever epidemics in 1990s West Africa, where conflict-driven vaccination lapses triggered outbreaks (4). Overall, the data affirm that conflict strongly correlates with immunization failure and disease resurgence.

Conflict-displaced children, whether internally or as refugees, face major immunization gaps. Several studies report lower vaccination rates among refugee children versus host populations (15–17), showing that conflict impacts not only war-torn countries but also neighbors. Tailored interventions are needed in camps, especially during pandemics, with community volunteers playing a key role in sustaining care (27). Refugee children often fall short of WHO immunization targets, and overcrowding plus malnutrition amplify outbreak risks—measles mortality can be drastically higher in camps. These findings confirm that war-related migration and inequitable vaccine access are tightly linked, underscoring the urgent need to close these gaps. Similar concerns have been reported regarding recent migration flows from conflict areas in the Mediterranean region, impacting vaccination coverage in host countries (28).

The literature identifies key adaptive strategies to sustain immunization in conflict, notably immunization ceasefires—short-term truces that allow vaccination campaigns. These “days of tranquillity,” promoted by global actors, have proven effective in averting outbreaks (8). However, their fragility is evident: mistrust, insecurity, and politicized corridors hinder implementation. WHO emphasizes that while such pauses save lives, lasting immunization success requires peace. Ceasefires remain valuable but temporary solutions—not substitutes for stable health systems.

Beyond ceasefires, field innovations support vaccination in insecure areas. Following UNICEF guidelines, health workers have reached remote zones in Yemen and Mali on foot or mule, and women-led teams in Afghanistan have accessed households otherwise unreachable (29). Digital tools also aid in tracking vaccine logistics (21, 22). These strategies—mobile clinics, NGO support, electronic registries—show the adaptability of providers in crises. UNICEF promotes such flexible methods (e.g., solar cold chains, integrated services), though their success often depends on continued external support, which may falter in protracted conflicts.

This narrative review has several limitations. Its selective approach may introduce bias, and the lack of formal quality appraisal limits comparability. Study heterogeneity—across conflicts, outcomes, and methods—precludes uniform metrics. Data from war zones are often incomplete due to weak surveillance systems (4, 9), especially in areas under non-state control. Most research focuses on young children and a few core vaccines, with little data on adolescents, adults, or non-routine immunizations like HPV. Long-term impacts and recovery trajectories are also poorly documented. These gaps highlight the need for broader, higher-quality research in underrepresented contexts. Moreover, the review’s findings must be interpreted with caution due to the considerable heterogeneity among conflicts. Differences in conflict duration, intensity, and the demographics of affected populations can substantially influence vaccination outcomes. Most included studies focus on large-scale and well-documented conflicts such as those in Syria, Ukraine, and Nigeria, which may not be entirely representative of smaller, less visible, or under-reported conflicts. Thus, generalizability to all conflict settings is limited.

Despite limitations, the findings have major policy implications. Immunization should remain a humanitarian and public health priority during conflicts. Vaccinating during crises prevents outbreaks and is a recognized right in emergencies. Aid programs must integrate immunization into emergency responses, including stockpiling vaccines, reinforcing cold chains, and deploying rapid-response teams. Polio campaigns show it’s feasible to maintain coverage via negotiated access and community engagement. UN agencies and NGOs can act as neutral brokers to secure safe vaccination corridors. When access is blocked, vaccines should be offered at borders or checkpoints. Conflict-affected countries need flexible strategies—like working with local providers and training volunteers—while host countries should provide catch-up vaccines and integrate displaced children into national schedules, easing access with mobile clinics and simplified procedures.

Vaccination in conflict zones is essential to achieving global disease control goals. Armed conflicts also contribute to antibiotic resistance, requiring preventive action (30). Setbacks in immunization, especially among “zero-dose” children in fragile settings, threaten the success of efforts like Immunization Agenda 2030 (31). Donors must prioritize vaccines alongside food and water, investing in tools like GPS tracking and e-registries to sustain coverage during crises. Protecting health workers and preventing attacks on facilities is crucial, as is tackling misinformation through trusted messengers and clear communication. Ultimately, only peace can ensure lasting immunization. Rebuilding strong public health systems with robust surveillance is vital (32). While emergency measures help, they address symptoms, not causes. Securing vaccination in conflict is both a technical and moral imperative.

## Conclusion

This review confirms that armed conflicts substantially disrupt immunization systems, resulting in significant declines in vaccine coverage and increased outbreaks of VPDs, particularly affecting children and displaced populations. Conflict-affected regions disproportionately account for global VPD cases, highlighting that immunization must be maintained as an essential humanitarian priority during warfare. While strategies such as immunization ceasefires, innovative vaccine delivery methods, and community engagement show promise in temporarily mitigating immunization gaps, they remain fragile and reliant on external support. The review underscores an urgent need for comprehensive international support, including proactive contingency planning, strengthened infrastructure, and enhanced protection for health workers. Ultimately, achieving sustainable immunization coverage requires lasting peace and stable health systems, reinforcing the imperative that global peace-building and public health initiatives must be closely integrated.
